# Newly Discovered Antimicrobial Peptide Scyampcin_44–63_ from Scylla paramamosain Exhibits a Multitargeted Candidacidal Mechanism *In Vitro* and Is Effective in a Murine Model of Vaginal Candidiasis

**DOI:** 10.1128/aac.00022-23

**Published:** 2023-05-10

**Authors:** Ying Zhou, Xiangyu Meng, Fangyi Chen, Ming Xiong, Weibin Zhang, Ke-Jian Wang

**Affiliations:** a State Key Laboratory of Marine Environmental Science, College of Ocean and Earth Sciences, Xiamen University, Xiamen, China; b State-Province Joint Engineering Laboratory of Marine Bioproducts and Technology, College of Ocean and Earth Sciences, Xiamen University, Xiamen, China

**Keywords:** antimicrobial peptides, Scyampcin_44–63_, candidacidal mechanism, therapeutic effect, vulvovaginal candidiasis

## Abstract

The emergence of azole-resistant and biofilm-forming Candida spp. contributes to the constantly increasing incidence of vulvovaginal candidiasis. It is imperative to explore new antifungal drugs or potential substituents, such as antimicrobial peptides, to alleviate the serious crisis caused by resistant fungi. In this study, a novel antimicrobial peptide named Scyampcin_44–63_ was identified in the mud crab Scylla paramamosain. Scyampcin_44–63_ exhibited broad-spectrum antimicrobial activity against bacteria and fungi, was particularly effective against planktonic and biofilm cells of Candida albicans, and exhibited no cytotoxicity to mammalian cells (HaCaT and RAW264.7) or mouse erythrocytes. Transcriptomic analysis revealed four potential candidacidal modes of Scyampcin_44–63_, including promotion of apoptosis and autophagy and inhibition of ergosterol biosynthesis and the cell cycle. Further study showed that Scyampcin_44–63_ caused damage to the plasma membrane and induced apoptosis and cell cycle arrest at G_2_/M in C. albicans. Scanning and transmission electron microscopy demonstrated that Scyampcin_44–63_-treated C. albicans cells were deformed with vacuolar expansion and destruction of organelles. In addition, C. albicans cells pretreated with the autophagy inhibitor 3-methyladenine significantly delayed the candidacidal effect of Scyampcin_44–63_, suggesting that Scyampcin_44–63_ might contribute to autophagic cell death. In a murine model of vulvovaginal candidiasis, the fungal burden of vaginal lavage was significantly decreased after treatment with Scyampcin_44–63_.

## INTRODUCTION

Vulvovaginal candidiasis (VVC) is the most prevalent candidal infection, mostly occurring in women of childbearing age, and afflicts almost 75% of women at least once in their lifetime, among whom nearly half will experience a recurrence (1, 2). Furthermore, 5% to 8% of adult women suffer recurrent vulvovaginal candidiasis (RVVC), defined as four or more episodes annually. Although VVC is nonlethal, VVC causes discomfort, pain, and social embarrassment along with considerable economic loss, producing approximately $1.8 billion in medical costs and $1 billion in additional annual payments in the United States alone (1, 3). Nystatin- or azole-based topical agents are intravaginally applied to treat ordinary C. albicans VVC in the United States (3). Fluconazole has become the primary choice for treating VVC because of its high oral bioavailability, convenient drug delivery, and favorable safety properties. However, continuous use or inappropriate usage exacerbates the emergence of fluconazole-resistant C. albicans and has greatly hindered clinical therapy (4).

Antimicrobial peptides (AMPs) are considered promising alternatives to antibiotics, as AMPs have multiple modes of action, broad-spectrum activity against pathogens, and a low propensity to produce resistance (5, 6). Most antifungal peptides interact with fungal cell membranes, causing membrane disruption. Some antifungal peptides interfere with cell wall synthesis, while others exert different fungicidal mechanisms, e.g., stimulating reactive oxygen species (ROS) production, dissipating mitochondrial membrane potential, triggering apoptosis, disrupting cation homeostasis, inducing ATP efflux, interfering with the cell cycle, and disrupting autophagy and vacuolar function (5). According to the clinical data shown in the Data Repository of Antimicrobial Peptides (http://dramp.cpu-bioinfor.org/, accessed on 21 November 2022), 77 AMPs are under clinical trials. However, most of these AMPs have been shown to target against bacterial infections, while only a few are applied for treating candidiasis, including PAC-113 and CZEN-002. Therefore, there is an urgent need to explore new antifungal compounds.

The mud crab Scylla paramamosain is a kind of marine crustacean that relies only on the effectors of innate immunity, such as AMPs, against invading pathogens. Frequent exposure to a complex and changing microbial environment makes S. paramamosain an arsenal of AMPs. Since 2006, our group has identified various novel AMPs in S. paramamosain, such as Scygonadin (7), Scyreprocin (8), Sparamosin_26–54_ (9), Sparanegtin (10). and Spampcin_56–86_ (11). In the present study, we identified a novel AMP named Scyampcin_44–63_ from the mud crab S. paramamosain. Considering that C. albicans is the most prevalent human fungal pathogen and that Scyampcin_44–63_ has potent anti-candidal activity, we performed RNA sequencing (RNA-seq) to reveal the potential candidacidal mechanism of Scyampcin_44–63_ and verified it in experiments. We further evaluated the possibility of clinical application by exploring the efficacy of Scyampcin_44–63_ *in vivo*.

## RESULTS

### Cloning and bioinformatics analysis.

Based on the transcriptional database of S. paramamosain established by our research group, we screened and cloned a new functional gene that had no similarity in nucleotide or amino acid sequence with the existing online database, and named it Scyampcin (GenBank accession number MW388710). The full-length cDNA of Scyampcin consists of a 5′-untranslated region (UTR) of 132 bp, an open reading frame (ORF) of 213 bp, and a 3′-UTR of 144 bp (excluding the poly[A] tail) (Fig. S1A). Scyampcin encodes 70 amino acid residues, with a calculated mass of 8.2 kDa and an estimated isoelectric point (pI) of 10.11. The predicted secondary and tertiary structures showed that Scyampcin contained α-helices and β-sheets (Fig. S1B and C). According to the predicted antimicrobial region within peptides in the CAMP_R3_ database, we synthesized the C-terminal amidated truncated peptide Scyampcin_44–63_. The physicochemical parameters of Scyampcin_44–63_ are shown in Fig. S1D. Scyampcin_44–63_ contains 20 amino acids and is rich in lysine (Lys, 30%) and phenylalanine (Phe, 15%), with a hydrophobic ratio of 21.1% and a net charge of +6.

### Scyampcin_44–63_ exhibits potent antimicrobial activity.

The antimicrobial activities of Scyampcin_44–63_ were determined as shown in Table 1. Scyampcin_44–63_ showed broad-spectrum antimicrobial activity against Gram-positive bacteria (Staphylococcus aureus, Bacillus subtilis, Bacillus cereus, Staphylococcus epidermidis, Listeria monocytogenes, Enterococcus faecium, and Enterococcus faecalis), Gram-negative bacteria (Shigella flexneri, Acinetobacter baumannii, Escherichia coli, and Pseudomonas aeruginosa), and fungi (Cryptococcus neoformans, Candida albicans, Candida tropicalis, Candida parapsilosis, Candida krusei, Fusarium graminearum, and Fusarium solani). The minimum bactericidal concentration (MBC) or minimum fungicidal concentration (MFC) values of Scyampcin_44–63_ for all of the tested microorganisms were below 6 μM, except for the MBC of 12 to 24 μM for P. aeruginosa. Even when the concentrations of Scyampcin_44–63_ reached 96 μM, Scyampcin_44–63_ was safe for mammalian HaCaT or RAW264.7 cells and was without hemolytic activity toward mouse erythrocytes (Fig. S2).

**TABLE 1:** Antimicrobial activities of Scyampcin_44–63_^*a*^

Microorganisms	CGMCC no.	MBC or MFC (μM)
Gram-positive bacteria		
Staphylococcus aureus	1.2465	3 to 6
Bacillus subtilis	1.3358	0 to 3
Bacillus cereus	1.3760	3 to 6
Staphylococcus epidermidis	1.4260	0 to 3
Listeria monocytogenes	1.10753	3 to 6
Enterococcus faecium	1.131	0 to 3
Enterococcus faecalis	1.2135	3 to 6
Gram-negative bacteria		
Shigella flexneri	1.1868	0 to 3
Acinetobacter baumannii	1.6769	0 to 3
Escherichia coli	1.2389	0 to 3
Pseudomonas aeruginosa	1.1785	12 to 24
Fungi		
Cryptococcus neoformans	2.1563	0 to 3
Candida albicans	2.2411	3 to 6
Candida tropicalis	2.1975	0 to 3
Candida parapsilosis	2.1846	0 to 3
Candida krusei	2.1875	3 to 6
Fusarium graminearum	3.4521	3 to 6
Fusarium solani	3.584	3 to 6

a CGMCC, China General Microbiological Culture Collection Center; MBC, minimum bactericidal concentration; MFC, minimum fungicidal concentration.

### Transcriptomic analysis of C. albicans.

**FIG 1:**
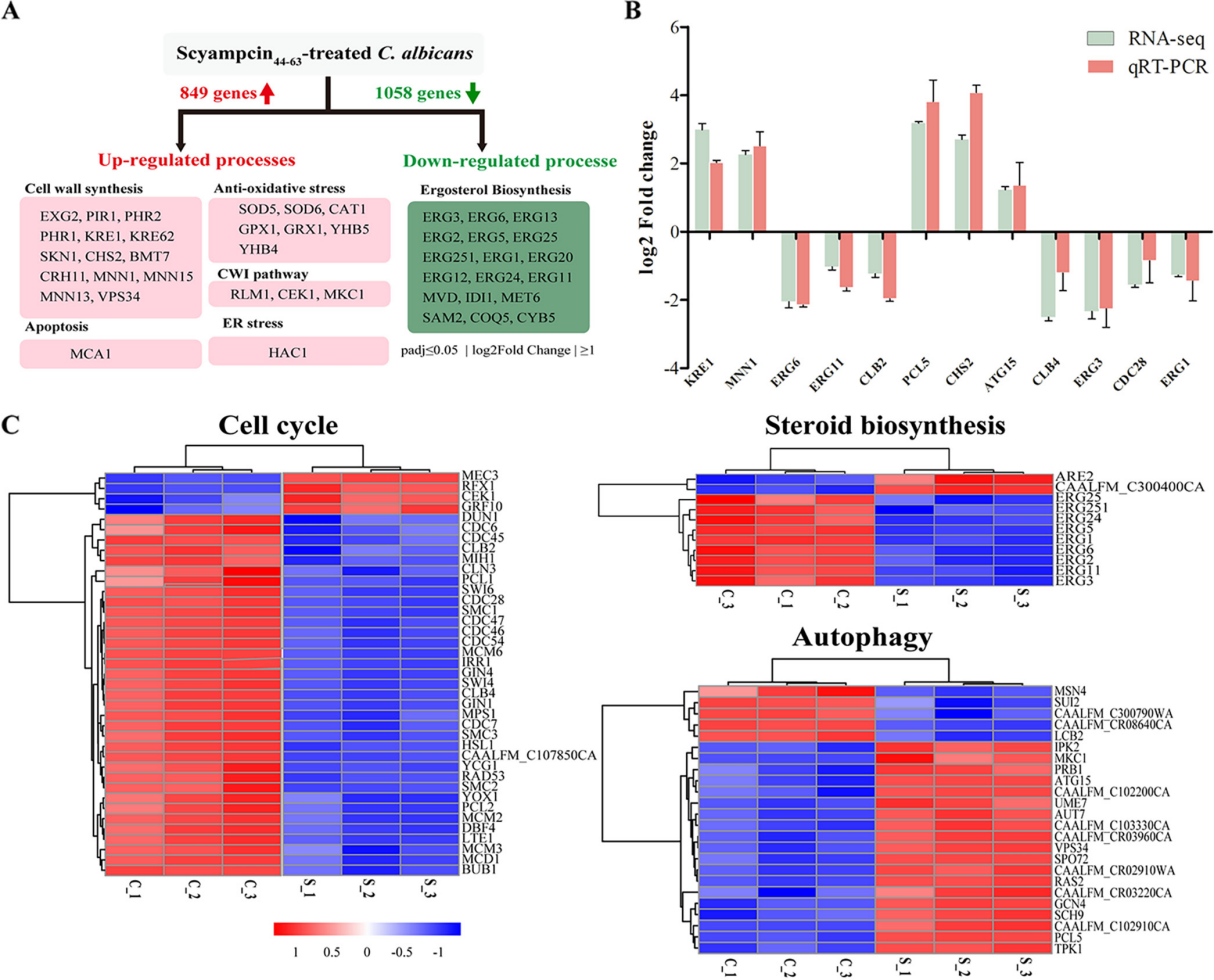
Transcriptomic analysis of Scyampcin_44–63_-treated C. albicans. (A) Analysis of DEGs in C. albicans after Scyampcin_44–63_ treatment. DEGs, differentially expressed genes. (B) Comparison of expression levels between RNA sequencing (RNA-seq) and quantitative reverse-transcription PCR (qRT-PCR). (C) Hierarchical clustering analysis of several enriched pathways. C. albicans cells were untreated or treated with Scyampcin_44–63_ for 1 h, and RNA sequencing was performed. C_1, C_2, and C_3 represent three independent treatments of the control, and S_1, S_2, and S_3 represent three independent treatments of Scyampcin_44–63_. Colored bars indicate the Z score, which was the normalized value of differential gene *FPKM*. The deeper red shades indicate higher expression levels, while the deeper blue shades indicate lower expression levels.

**(i) Analysis and verification of differentially expressed genes (DEGs).** We performed a transcriptomic analysis of untreated and Scyampcin_44–63_-treated C. albicans to gain further insight into the candidacidal mechanism of Scyampcin_44–63_. After Scyampcin_44–63_ treatment, a total of 1,907 DEGs were obtained in C. albicans, including 849 upregulated genes and 1,058 downregulated genes (Fig. 1A). The upregulated genes were involved in different biological processes, such as cell wall synthesis, the cell wall integrity (CWI) pathway, antioxidative stress, endoplasmic reticulum (ER) stress, and apoptosis (Fig. 1A; Table S4). The downregulated genes mainly related to ergosterol biosynthesis (Fig. 1A; Table S5). To validate the RNA-seq results, we randomly selected 12 genes, including five upregulated and seven downregulated genes, from the DEG libraries for quantitative reverse-transcription PCR (qRT-PCR) (primers for qRT-PCR are listed in Table S3). The trends of gene expression levels were consistent between qRT-PCR and RNA-seq. Hence, the qRT-PCR results validated the reliability of the RNA-seq data (Fig. 1B).

**(ii) KEGG enrichment analysis of DEGs.** In response to Scyampcin_44–63_ treatment, the KEGG enrichment analysis showed that pathways associated with amino acid metabolism and biosynthesis, biosynthesis of secondary metabolites, 2-oxocarboxylic acid metabolism, autophagy, glycerolipid metabolism, and inositol phosphate metabolism were upregulated, while pathways related to replication and repair, transcription, translation, cell cycle, meiosis, and steroid biosynthesis were downregulated (Table 2). The DEGs enriched in KEGG pathways (autophagy, cell cycle and steroid biosynthesis, corrected *P* values < 0.05, and |log_2_[fold change]| > 1) were individually subjected to hierarchical clustering analysis, and a heat map was constructed (Fig. 1C). Among these DEGs, 19 of the 25 genes participating in the autophagy pathway were upregulated, and 35 of the 39 genes related to the cell cycle and 9 of the 11 genes associated with steroid biosynthesis were downregulated.

**TABLE 2:** Significant enriched KEGG pathways of DEGs^*a*^

ID	Description	Input no.	Background no.	Corrected *P* value
Upregulated				
cal01230	Biosynthesis of amino acids	29	107	1.39 × 10^−5^
cal01210	2-Oxocarboxylic acid metabolism	13	32	1.64 × 10^−4^
cal00220	Arginine biosynthesis	8	16	1.23 × 10^−3^
cal00290	Valine, leucine, and isoleucine biosynthesis	7	13	1.23 × 10^−3^
cal00340	Histidine metabolism	7	13	1.23 × 10^−3^
cal04138	Autophagy: yeast	19	76	1.23 × 10^−3^
cal01110	Biosynthesis of secondary metabolites	50	322	4.80 × 10^−3^
cal00561	Glycerolipid metabolism	9	27	7.44 × 10^−3^
cal00562	Inositol phosphate metabolism	7	22	3.10 × 10^−2^
cal04136	Autophagy: other	7	22	3.10 × 10^−2^
Downregulated				
cal03010	Ribosome	96	119	7.67 × 10^−44^
cal03030	DNA replication	31	34	1.66 × 10^−16^
cal03430	Mismatch repair	16	21	3.65 × 10^−6^
cal03008	Ribosome biogenesis in eukaryotes	34	72	1.94 × 10^−5^
cal03410	Base excision repair	13	18	9.60 × 10^−5^
cal03440	Homologous recombination	13	20	4.66 × 10^−4^
cal00230	Purine metabolism	24	52	7.38 × 10^−4^
cal04111	Cell cycle: yeast	35	98	8.50 × 10^−3^
cal03420	Nucleotide excision repair	16	38	3.09 × 10^−2^
cal03020	RNA polymerase	13	29	3.43 × 10^−2^
cal04113	Meiosis: yeast	30	89	3.88 × 10^−2^
cal00100	Steroid biosynthesis	9	18	4.71 × 10^−2^

a The corrected *p* values were adjusted using the Benjamini and Hochberg method, which helped to better control the false-positive rate of the multiple hypothesis test. DEG, differentially expressed gene.

### Candidacidal and anti-biofilm activity of Scyampcin_44–63_.

**FIG 2:**
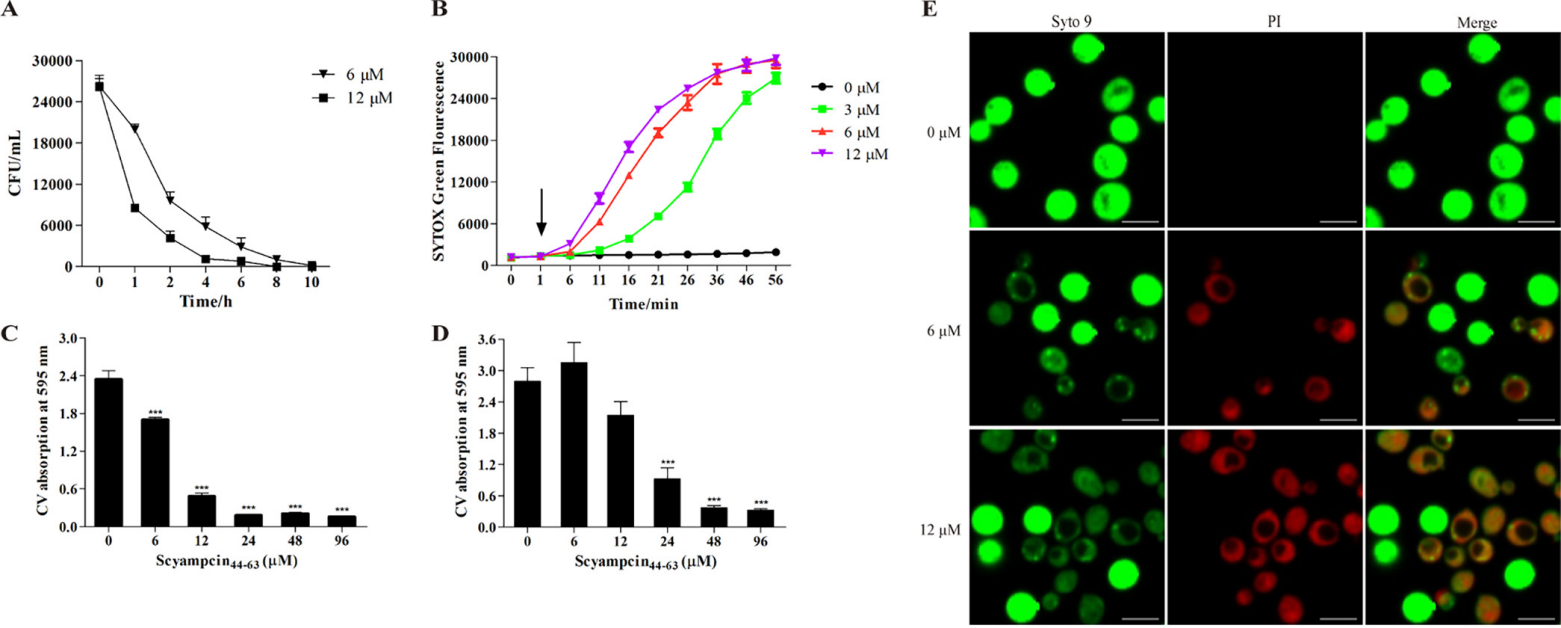
The candidacidal, anti-biofilm, and membrane permeability activities of Scyampcin_44–63_. (A) Time-killing kinetics of Scyampcin_44–63_ on C. albicans. (B) Scyampcin_44–63_ enhanced SYTOX green fluorescence intensity. The arrow indicates the time of peptide addition. (C) Scyampcin_44–63_ inhibited biofilm formation of C. albicans. (D) Scyampcin_44–63_ was effective against preformed biofilm of C. albicans. (A to D) Representative results of three repeats are shown, and error bars represent standard errors of the means (*n* = 3). (E) Scyampcin_44–63_ destroyed the membrane of C. albicans. C. albicans was exposed to Scyampcin_44–63_ for 30 min and then stained with propidium iodide (PI)/Syto 9 for 15 min. The samples were imaged by confocal laser microscopy. The bars at the bottom right of each picture indicate 5 μm. Asterisks indicate significant difference: *, *P < *0.05; **, *P < *0.01; ***, *P < *0.001. Significant difference was compared with vehicle control (0 μM in the images).

As Scyampcin_44–63_ had potent candidacidal activity, we examined the time-killing curve and the anti-biofilm activity. The candidacidal activity of Scyampcin_44–63_ was determined by colony count assays (Fig. 2A). After exposure to 6 μM and 12 μM Scyampcin_44–63_ for 1 h, 24.1% and 67.4% of C. albicans died, respectively. Scyampcin_44–63_ at a dose of 6 μM killed 99.4% of C. albicans within 10 h, and 12 μM Scyampcin_44–63_ killed 99.9% of C. albicans in 8 h (Fig. 2A). Exogenous ergosterol had less of an effect on Scyampcin_44–63_ (MIC up to 2-fold) than amphotericin B (AMB) (MIC up to 8-fold) against C. albicans (Table S6). As Fig. S5 shown, the proportion of propidium iodide (PI) in C. albicans were decreased from 24.2% to 7.73%, 5.29%, and 4.98% when pretreated Scymapcin_44–63_ with 100, 200, and 400 μg/mL of ergosterol, respectively. Scyampcin_44–63_ at a dose ≥6 μM significantly inhibited the biofilm formation of C. albicans (Fig. 2C), while ≥24 μM Scyampcin_44–63_ was effective against preformed biofilms of C. albicans (Fig. 2D). We further verified that Scyampcin_44–63_ was also effective against hyphal forms of C. albicans (Fig. S3). The amphiphilic structure allows AMPs to interact with microbial membranes, causing membrane permeabilization, which is the most common microbicidal mechanism of AMPs. Thus, we used the fluorescent dyes SYTOX Green and PI/Syto 9 to evaluate the membrane permeability of C. albicans cells caused by Scyampcin_44–63_. SYTOX Green and PI can cross the damaged membrane and excite green and red fluorescence after binding to nucleic acids, respectively. The fluorescence intensity of SYTOX Green in C. albicans was enhanced after exposed to different concentrations of Scyampcin_44–63_ with concentration and time dependence (Fig. 2B). Scyampcin_44–63_ promoted the uptake of PI by C. albicans and decreased the fluorescence intensity of Syto 9, indicating that the membrane of C. albicans was disrupted by Scyampcin_44–63_ (Fig. 2E). In addition, the cells treated with Scyampcin_44–63_ appeared smaller than those in the control group, and vacuolar expansion was observed (Fig. 2E). Furthermore, we confirmed this finding with flow cytometry. The forward scatter (FSC) was indeed decreased, which indicated cell shrinkage. Cell shrinkage occurred prior to membrane permeabilization (Fig. S4). The aforementioned results evidently showed that Scyampcin_44–63_ caused membrane permeabilization, inhibited the biofilm formation, and was effective against preformed biofilms of C. albicans.

### Scyampcin_44–63_ induces apoptosis in C. albicans.

**FIG 3:**
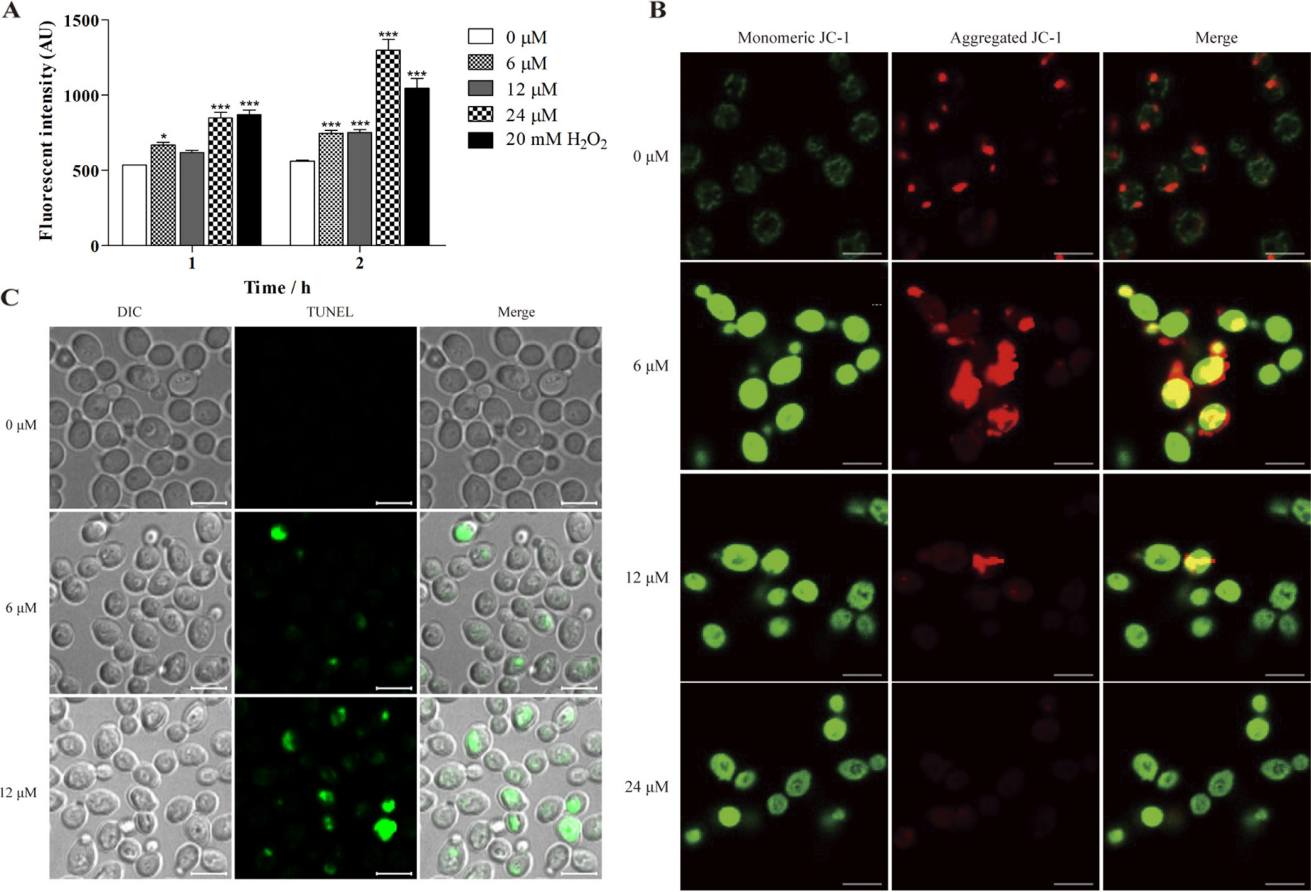
Scyampcin_44–63_ triggered apoptosis of C. albicans. (A) Intracellular reactive oxygen species (ROS) levels. C. albicans cells (2.5 × 10^6^ cells/mL) were pretreated with the 2′,7′-dichlorofluorescin diacetate (DCFH-DA) probe for 30 min, and then the excessive stain was removed. The results were recorded with a multimode microplate reader (excitation/emission = 485 nm/525 nm) after incubation with Scyampcin_44–63_ or H_2_O_2_ (as a positive control) for 1 and 2 h. Asterisks indicate significant difference: *, *P < *0.05; **, *P < *0.01; ***, *P < *0.001, compared with control. Representative results of three repeats are shown, and error bars represent standard errors of the means (*n* = 3). (B) Scyampcin_44–63_ destroyed ΔΨm. (C) DNA fragmentation caused by Scyampcin_44–63_. Representative results of three repeats are shown. (B, C) C. albicans cells (2.5 × 10^6^ cells/mL) exposed to Scyampcin_44–63_ for 2 h. JC-1 and terminal deoxynucleotidyltransferase-mediated dUTP-biotin nick end labeling (TUNEL) staining were performed. The samples were observed by confocal laser microscopy. The bars on the bottom right of each picture indicate 5 μm. DIC, differential inference contrast.

Transcriptomic analysis showed that Scyampcin_44–63_ upregulated genes related to antioxidative stress and apoptosis, which indicated that Scyampcin_44–63_ might cause oxidative damage and trigger apoptosis. We measured intracellular reactive oxygen species (ROS) levels by using an oxidant-sensitive dye, 2′,7′-dichlorofluorescin diacetate (DCFH-DA), which turns into 2′,7′-dichlorofluorescein (DCF) with strong fluorescence intensity after oxidation. In this study, the fluorescence intensity was significantly enhanced by Scyampcin_44–63_ or H_2_O_2_, indicating the accumulation of ROS in C. albicans (Fig. 3A). Scyampcin_44–63_ induced the generation of ROS in a concentration-dependent manner, while 10 mM H_2_O_2_ generated less ROS than 24 μM Scyampcin_44–63_ at 2 h (Fig. 3A). ROS are mainly produced in mitochondria and cause damage to the mitochondrial membrane by dissipating its membrane potential (12). Thus, we used JC-1 to monitor the changes in mitochondrial membrane potential. In normal cells, JC-1 aggregates in the mitochondrial matrix and exhibits red fluorescence. When the mitochondrial membrane potential (ΔΨ_m_) dissipates, JC-1 aggregates can be converted to JC-1 monomers (green fluorescence). After 2 h of treatment with 6 μM Scyampcin_44–63_, some cells presented hyperpolarized mitochondrial membrane potential, while others completely lost mitochondrial membrane potential (Fig. 3B). Scyampcin_44–63_ stimulated the generation of ROS and destroyed the mitochondrial membrane potential of C. albicans. Excessive ROS induce oxidative damage to important cellular components, such as proteins, lipids, or nucleic acids, and can trigger apoptosis (13). Furthermore, we used terminal deoxynucleotidyltransferase-mediated dUTP-biotin nick end labeling (TUNEL) staining to determinate whether Scyampcin_44–63_ induced apoptosis in C. albicans. Both 6 μM and 12 μM Scyampcin_44–63_ increased green fluorescence in C. albicans, indicating the occurrence of DNA fragmentation (Fig. 3C), which is a sign of apoptosis.

### Scyampcin_44–63_ induces cell cycle arrest at G_2_/M phase.

**FIG 4:**
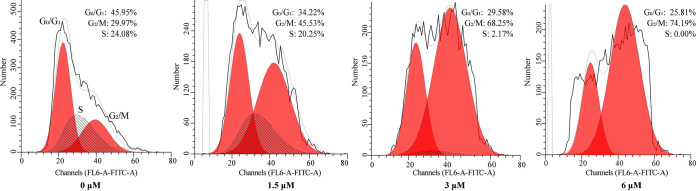
The effect of Scyampcin_44–63_ on the cell cycle of C. albicans. C. albicans were collected after exposure to different concentrations of Scyampcin_44–63_ and then fixed with precooled 70% ethanol. The fixed cells were washed with sodium citrate buffer twice, treated with RNase A for 1 h, and stained with SYTOX Green for 30 min. The DNA contents were measured with a flow cytometer. Representative results of three repeats are shown.

Several reports have shown that apoptosis is closely associated with cell cycle arrest, and the cell cycle of Scyampcin_44–63_-treated C. albicans is one of the pathways significantly downregulated in KEGG enrichment analysis. Therefore, we detected the cell cycle of C. albicans after exposure to different concentrations of Scyampcin_44–63_. Compared with the untreated group, after exposure to 3 μM and 6 μM Scyampcin_44–63_, the proportions of G_2_/M phase cells (from 24.08% to 68.25% and 74.19%, respectively) was increased, and both the G_1_/G_0_ phase (from 45.95% to 29.58% and 25.81%, respectively) and S phase proportions decreased (from 24.08% to 2.17% and 0.00%, respectively) (Fig. 4), indicating that the cell cycle of C. albicans was arrested at the G_2_/M phase by Scyampcin_44–63_.

### Morphological change and extracellular DNA concentration.

**FIG 5:**
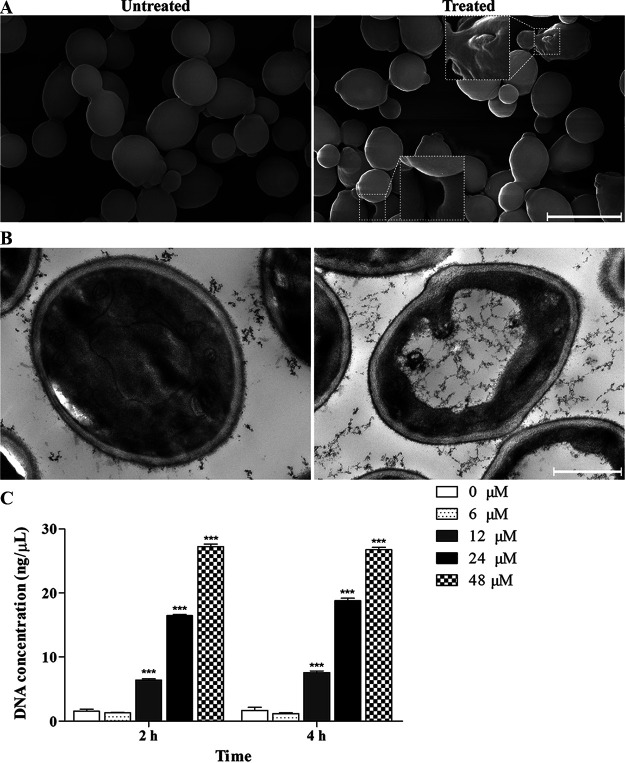
Morphological changes and extracellular DNA concentration of C. albicans induced by Scyampcin_44–63_. (A, B) Morphological changes of C. albicans induced by Scyampcin_44–63_. After incubation with 6 μM Scyampcin_44–63_ for 1 h, the samples were subjected to SEM or TEM methods and photographed by scanning (A) and transmission (B) electron microscopy. The bars on the bottom right of the picture indicate 5 μm (A) and 1 μm (B). (A) Dotted boxes represent zoomed-in portions of samples. Representative results of three repeats are shown. (B) Representative images of two repeats. (C) Extracellular DNA concentration of C. albicans after treatment with Scyampcin_44–63_. The supernatants of C. albicans were collected to detect the DNA concentration after exposure to Scyampcin_44–63_. Representative results of three repeats are shown, and error bars represent standard errors of the means (*n* = 3). Asterisks indicate significant difference: *, *P < *0.05; **, *P < *0.01; ***, *P < *0.001, compared with controls.

To explore the anti-candidal mechanism of Scyampcin_44–63_, we used scanning electron microscopy (SEM) and transmission electron microscopy (TEM) to observe the morphological and ultrastructural changes of C. albicans induced by Scyampcin_44–63_, respectively. The untreated yeasts were round, while the Scyampcin_44–63_-treated yeasts were deformed and dented (Fig. 5A). TEM images showed that the cell wall was deformed, the membrane was dented and the vacuole was expanded in Scyampcin_44–63_-treated yeasts. There were obvious organelle boundaries in the untreated yeasts, meanwhile, in the Scyampcin_44–63_-treated yeasts, the organelles were barely visible (Fig. 5B). In addition, we measured the extracellular DNA concentration of C. albicans to check whether damaged nuclear and plasma membranes induced the leakage of DNA. The results showed that the concentrations of extracellular DNA in C. albicans were significantly elevated when exposed to 12 to 48 μM Scyampcin_44–63_ for 2 h and 4 h (Fig. 5C).

### 3-MA delayed the candidacidal effect of Scyampcin_44–63_.

**FIG 6:**
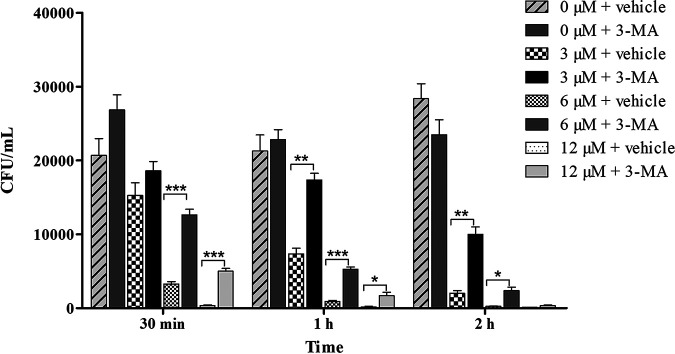
3-Methyladenine (3-MA) significantly delayed the candidacidal rate of Scyampcin_44–63_. C. albicans was pretreated with or without 10 mM 3-MA for 1 h. Different concentrations of Scyampcin_44–63_ were added and incubated for 30 min, 1 h, and 2 h. The mixed suspensions were diluted and spread onto yeast extract peptone dextrose agar plates (*n* = 3). Asterisks indicate significant difference: *, *P < *0.05; **, *P < *0.01; ***, *P < *0.001. Significant differences were analyzed between the same concentration of Scyampcin_44–63_ with and without (vehicle control) treatment with 3-MA. Representative results of two repeats are shown, and error bars represent standard errors of the means (*n* = 3).

We observed vacuolar expansion in C. albicans after treatment with Scyampcin_44–63_ (Fig. 2E and 5B), and the autophagy pathway was one of the significantly upregulated pathways upon KEGG enrichment analysis (Table 2). Thus, we evaluated the effect of the autophagy inhibitor 3-methyladenine (3-MA) on the candidacidal activity of Scyampcin_44–63_. 3-MA significantly delayed the fungicidal rate of 3 μM Scyampcin_44–63_ against C. albicans at 1 h and 2 h. 3-MA significantly decreased the candidacidal activity of 6 μM Scyampcin_44–63_ at 30 min, 1 h, and 2 h. The remarkable influence of 3-MA on anti-candidal activity of 12 μM Scyampcin_44–63_ occurred at 30 min and 1 h (Fig. 6).

### Topical treatment with Scyampcin_44–63_ decreased the fungal burden *in vivo*.

**FIG 7:**
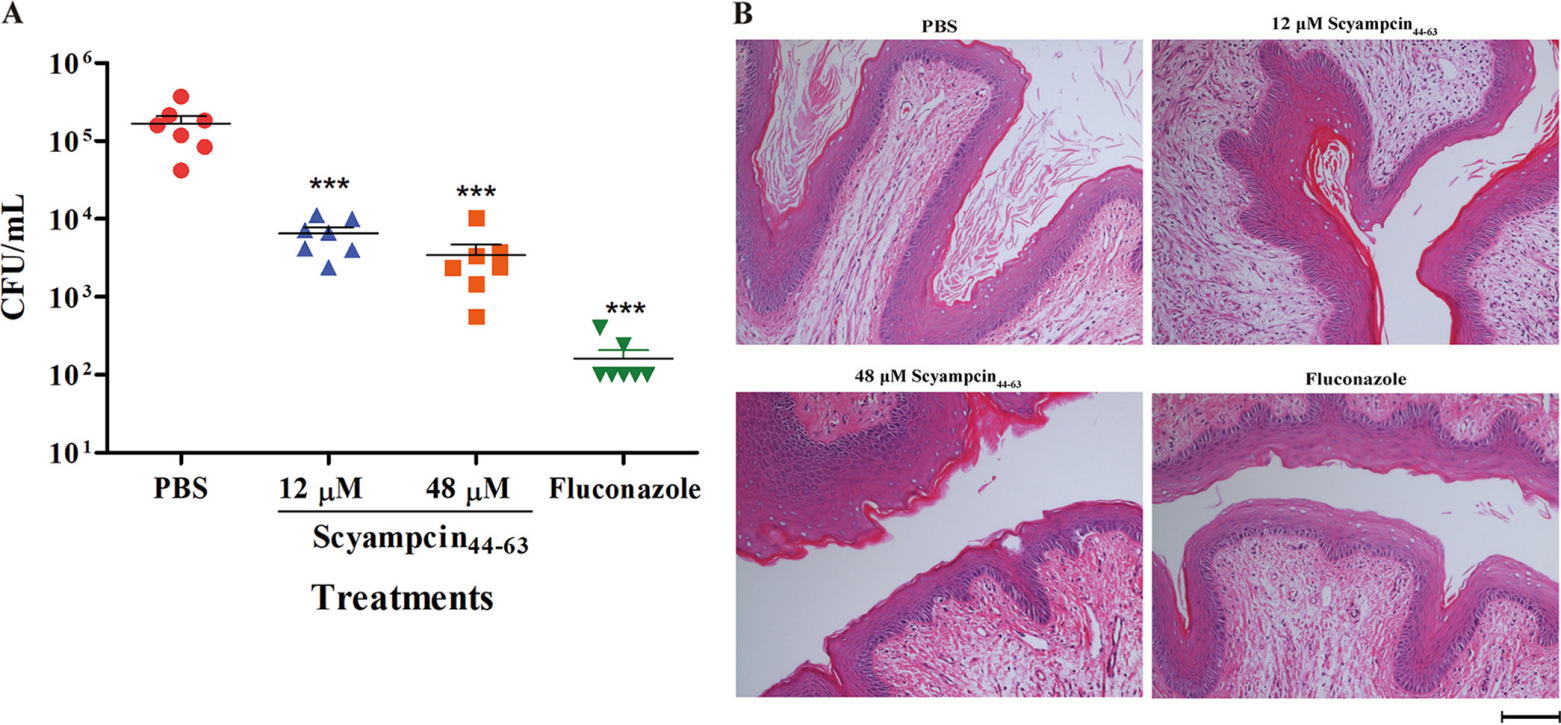
Efficacy of Scyampcin_44–63_ as the topical treatment for murine vulvovaginal candidiasis (VVC). (A) The fungal burden in vaginal lavage fluid. Representative results of two repeats are shown, and error bars represent standard errors of the means (*n* = 7), *** indicates *P* < 0.001. (B) Histological analysis of vaginal tissues with hematoxylin and eosin (HE) staining. The bar at the bottom right of the picture indicates 100 μm. Each group of mice (*n* = 7) continuously received treatment with phosphate-buffered saline (PBS), 12 μM Scyampcin_44–63_, 48 μM Scyampcin_44–63_, or 100 μg/mL fluconazole for 3 days (at intervals of 12 h) 24 h postinfection.

Because Scyampcin_44–63_ possessed potent anti-candidal activity *in vitro*, we further evaluated the safety and antifungal effect of Scyampcin_44–63_ in a murine model of VVC. Compared with the control (phosphate-buffered saline [PBS]) group, intravaginal treatment with Scyampcin_44–63_ (12 μM and 48 μM) and fluconazole (100 μg/mL) did not cause any significant change in lactate dehydrogenase (LDH) levels, and both were relatively safe for mice (Fig. S6). Topical treatment with Scyampcin_44–63_ or fluconazole significantly decreased the fungal burden in vaginal lavage fluid (Fig. 7A). The corresponding mean CFU levels were 1.67 × 10^5^ for the PBS group and 6.5 × 10^3^, 3.4 × 10^3^, and 1.63 × 10^3^ CFU/mL for the 12 μM Scyampcin_44–63_, 48 μM Scyampcin_44–63_, and 100 μg/mL fluconazole treatment groups, representing 96.1%, 97.9%, and 99.9% inhibition of vaginal colonization, respectively. Consistent with the fungal burden results, fewer pseudohyphae were observed after treatment with Scyampcin_44–63_ or fluconazole than in the PBS group (Fig. 7B).

## DISCUSSION

C. albicans is responsible for 90% of VVC cases, afflicting 75% of women globally (1). With the increasing incidence of azole-resistant and biofilm-forming Candida species, there is an urgent need to explore new antifungal drugs (3, 14). In the present study, we identified a novel AMP with broad-spectrum antimicrobial activity named Scyampcin_44–63_ from the mud crab S. paramamosain. Scyampcin_44–63_ inhibited C. albicans planktonic growth and biofilm formation and effectively inhibited preformed biofilms, without cytotoxicity toward mammalian cells (HaCaT and RAW264.7) or mouse erythrocytes. Thus, we performed RNA-seq to analyze the potential candidacidal mechanism of Scyampcin_44–63_; the results suggest that Scyampcin_44–63_ might inhibit ergosterol biosynthesis, trigger autophagic cell death and apoptosis, and interfere with the cell cycle. Following these clues, we designed experiments to verify the antifungal features and mechanism and further evaluated the efficacy of Scyampcin_44–63_ *in vivo*.

Ergosterol is a vital component of the fungal plasma membrane that affects membrane permeability and the activity of membrane-bound enzymes and is also an important target for many antifungal drugs, such as azole and AMB (15). Consistent with the treatment with AMB (16) or MAF-1A (17), several ergosterol biosynthesis-related genes were downregulated in Scyampcin_44–63_-treated C. albicans. We demonstrated that the plasma membrane permeability of C. albicans was constantly increasing in response to Scyampcin_44–63_. Exogenous ergosterol decreased the activity of AMB or Scyampcin_44–63_ against C. albicans, indicating that Scyampcin_44–63_ might partially influence ergosterol biosynthesis and cause membrane damage in C. albicans.

Autophagy is an adaptation to cellular stress through engulfing damaged organelles and cytoplasmic components and degrading them within the lysosome/vacuole compartment (18). Excessive levels of autophagy contribute to autophagic cell death, which is accompanied by large-scale autophagic vacuolization in the cytoplasm (19). In the present study, the KEGG-enriched autophagy pathway of C. albicans was shown to be upregulated in response to Scyampcin_44–63_. TEM images showed that untreated cells were round and that organelles were clearly presented in C. albicans, while Scyampcin_44–63_-treated C. albicans cells exhibited deformed morphology, vacuolar expansion, and barely visible organelles. The autophagy inhibitor 3-MA significantly delayed the killing effect of Scyampcin_44–63_ against C. albicans. Thus, we speculated that Scyampcin_44–63_ caused organelle damage, resulting in the upregulation of autophagy-related genes and the induction of vacuolar expansion, which might ultimately trigger autophagic cell death in C. albicans. However, the underlying mechanism still needs further investigation.

Unlike autophagic cell death, intrinsic apoptosis has been widely demonstrated in Candida species (20). AMPs such as cecropin A (21) and psacotheasin (22) induce apoptosis in C. albicans. Intrinsic apoptosis is accompanied by the accumulation of ROS and mitochondrial dysfunction. ROS are by-products of cellular metabolism (13). Excessive ROS cause irreversible damage to proteins, lipids, nucleic acids, membranes, and organelles (22). Recently, many reported AMPs have been shown to induce cell death by generating ROS, such as plant defensins (NaD1, RsAFP2, HsAFP1, and PvD1) (22–27). Similarly, Scyampcin_44–63_ significantly increased the intracellular ROS level in C. albicans. As mitochondria are both the source and target of intracellular ROS, and mitochondrial membrane potential is indispensable for maintaining mitochondrial functions, we used the JC-1 dye to demonstrate that Scyampcin_44–63_ destroyed the mitochondrial membrane potential of C. albicans. Consistent with the process of hyperpolarization of the mitochondrial membrane potential induced by apoptotic agents (28), some cells presented hyperpolarization after exposure to 6 μM Scyampcin_44–63_, which occurs before total loss of the mitochondrial membrane potential and cell death. Increased ROS and destroyed mitochondrial membrane potential are markers of apoptosis, and we further verified that Scyampcin_44–63_ indeed triggered apoptosis by causing DNA fragmentation in C. albicans.

Several reports have shown that apoptosis is closely associated with cell cycle arrest; for example, antifungal natural products and their derivatives cause cellular apoptosis and dysregulate the cell cycle (29). The plant defensin HsAFP1 also causes S. cerevisiae cell cycle arrest at the G_2_/M phase (30). AMB (4 to 8 μg/mL) (31) and an antifungal compound citral derivative (29) cause cell cycle arrest and apoptosis in C. albicans. The antimicrobial peptides APP and BUF (5–21) cause C. albicans cell cycle arrest in the S phase and subsequently inhibit normal processes (32). However, the precise link between the cell cycle and apoptosis in C. albicans remains unclear. In the present study, several cell cycle- and mitosis-related genes of C. albicans were downregulated after Scyampcin_44–63_ treatment, and we demonstrated that Scyampcin_44–63_ induced cell cycle arrest at the G_2_/M phase in C. albicans, suggesting that one of the candidacidal mechanisms of Scyampcin_44–63_ is interference with cell proliferation and division.

The murine model of VVC has been well established, and it is helpful to evaluate the efficacy of antifungal agents, which is an indispensable step for future applications. Cytokines, antibodies, probiotics, and AMPs play vital roles in the immune defense of vaginal tissues against the invasion of Candida species (33). Several AMPs, such as gomesin (34), Hst-5 (35), and NFAP2 (3) significantly reduce the number of C. albicans cells in a murine model of VVC, even though the vaginal fungal burden is still present. Similarly, in this study, intravaginal administration of Scyampcin_44–63_ was effective in reducing the number of C. albicans cells, and fewer pseudohyphae were observed in the murine vagina, which could not eliminate the vaginal fungal burden to undetectable levels. Although AMPs are considered promising new antibiotic candidates, there are still some issues (low stability and bioavailability, potential toxicity, and high cost) that need to be solved for their application in the clinic (36). Some AMPs (such as LL-37 and β-defensins) are severely dysfunctional under physiological salt concentrations and are affected by host factors (37). These findings may partially explain why AMPs cannot fully eradicate the vaginal fungal burden in the murine model of VVC. Furthermore, C. albicans is a polymorphic fungus that grows as yeast, pseudohyphae, and true hyphae (38). C. albicans has the chance to convert yeast to hyphae once in contact with host cells, and the hyphae are more resistant to antifungal drugs, which greatly hampers treatment *in vivo* (39). Seeking methods to improve antimicrobial activity and safety *in vivo* requires further research. Previous studies have shown that some AMPs act synergistically with AMPs, surface-active agents, and traditional antibiotics, and these synergistic effects enhance their antimicrobial effect (36). For example, NFAP2 combined with fluconazole indeed enhances the efficacy *in vivo* (3). Loading AMPs into systems based on nanoparticles or microparticles with a targeted delivery capacity is another strategy to overcome the shortcomings of AMPs (36). In the present study, we provide an option for topical treatment of murine VVC with Scyampcin_44–63_, which needs further optimization. In the future, we will attempt to explore the synergistic effect of Scyampcin_44–63_ with AMPs or antibiotics and optimize the delivery approach to improve the efficacy of AMP *in vivo*.

## MATERIALS AND METHODS

### Scyampcin gene cloning.

The full-length cDNA of Scyampcin was amplified by rapid amplification of cDNA ends (RACE) PCR. The primers for RACE PCR were listed in Table S1. Mud crabs (S. paramamosain, weight: 300 ± 10 g) were obtained from Zhangpu FishFarm (Fujian, China), and tissues or organs were collected for further RNA extraction, respectively. TRIzol reagent (Invitrogen, USA), PrimeScript RT reagent kit with a genomic DNA (gDNA) eraser kit (TaKaRa, China), SMARTer RACE 5′/3′ kit were used to extract total RNA, synthesize cDNA, and RACE cDNA, respectively. The obtained fragment was cloned into pMD18-T Vector (TaKaRa, China) and sequenced by Bioray Biotechnology (Xiamen, China).

### Bioinformatics analysis and synthesis of Scyampcin_44–63_.

The homology and similarity of Scyampcin were analyzed at the National Center for Biotechnology Information (NCBI). The ORF was predicted by the orfinder tool of NCBI. The pI/*M*_w_ was computed by the Expasy tool. PSIPRED 4.0 and I-TASSER were used to predict secondary and tertiary structure, respectively. CAMP_R3_ was used to predict antimicrobial region within peptides. The aforementioned database or analysis software-related websites are listed in Table S2. Scyampcin_44–63_ (GKKKKRNMMKTKEPGIIFFF-NH_2_) was chemically synthesized by Genscript (Nanjing, China). The purity (≥95%) and molecular weight of both preparations were further confirmed by high performance liquid chromatography (HPLC) and mass spectrometry. The powdered peptide was stored at −80°C until use. The peptide was dissolved in sterile water and stored at −20°C.

### Strains and cultivation.

The strains used in antimicrobial activity assay were purchased from the China General Microbiological Culture Collection Center (CGMCC). Bacterial strains were cultivated in nutrient broth (NB) at 37°C, and fungal strains were cultured in yeast extract peptone dextrose (YPD) or potato dextrose agar (PDA) at 28°C. All experiments were strictly executed according to the guidelines of the standard biosecurity and institutional safety procedures established by Xiamen University.

### Cell line and cultivation.

Murine macrophage RAW264.7 was cultured in Dulbecco’s minimal essential medium (DMEM) containing 10% fetal bovine serum (FBS). Human immortalized keratinocytes HaCaT were maintained in minimal essential medium (MEM) with 10% FBS. The aforementioned cell lines were cultivated at 37°C with 5% CO_2_ atmosphere.

### Cytotoxicity assay.

The CellTiter 96 AQ*_ueous_* assay (Promega, USA) was used to determine cell viability (8). The CellTiter 96 AQ*_ueous_* Assay is composed of 3-(4,5-dimethylthiazol-2-yl)-5-(3-carboxymethoxyphenyl)-2-(4-sulfophenyl)-2H-tetrazolium (MTS) and phenazine methosulfate (PMS). RAW264.7 or HaCaT were seeded to 96-well plates (1 × 10^4^ cells/well) for 18 to 24 h. Fresh media with or without different concentrations of the test peptide were added slightly to the wells and incubated for another 24 h. The vehicle group was treated with fresh medium containing 5% water. After incubation with tested peptide, 20 μL of MTS-PMS reagent was added to each well and incubated for 1 to 4 h. The absorbance of each well was measured at 492 nm in a multimode microplate reader (Tecan, Switzerland). The experiments were repeated three times independently.

### Hemolytic activity assay.

The hemolytic activity of Scyapmcin_44–63_ on mouse erythrocytes was measured as previously reported (40). Briefly, and the erythrocytes were collected after washed three times with saline, resuspended in saline, and adjusted to 4% erythrocytes. The erythrocytes were incubated with different concentrations of Scyampcin_44–63_ for 1 h at 37°C. Treatment of saline (containing 5% water, as a vehicle control) and 1% Triton X-100 were used as negative and positive controls, respectively. The supernatants were centrifuged, their absorbance at 540 nm was measured using a multimode microplate reader (Tecan, Switzerland), and the ratio of hemolysis was calculated as previously described (40). The experiments were repeated three times independently.

### Antimicrobial activity assay.

The broth dilution method was used to determine the antimicrobial activity of the peptide based on a previous report with some modifications (9). Briefly, microorganisms were cultured to midlogarithmic phase and then harvested. The bacteria were diluted to approximately 5 × 10^5^ CFU/mL with Mueller-Hinton broth, and yeasts or conidia of filamentous fungi were diluted to approximately 5 × 10^4^ cells/mL in RPMI 1640 medium buffered with 0.165 mol/liter 3-morpholinopropane-1-sulfonic acid (pH of 7.0, referred to as RPMI-MOPS). Finally, the obtained microbial suspensions were mixed with serially diluted peptides in 96-well plates and then incubated under the corresponding conditions for 24 or 48 h. The final concentration contains 50% RPMI-MOPS (vehicle control). The MIC, MBC, and MFC values were recorded as previously described (9). The experiments were performed three times independently.

The effect of exogenous ergosterol on the MICs of Scyampcin_44–63_ or amphotericin B (AMB) was determined according to the aforementioned antimicrobial activity assay, but 50, 100, or 200 μg/mL ergosterol was added to RPMI-MOPS (Sigma-Aldrich, USA) (24). The experiments were repeated three times independently.

### Transcriptomic analysis.

C. albicans (3.75 × 10^7^ cells/mL in RPMI-MOPS) were incubated with an equal volume of Scyampcin_44–63_ maintained in shaking at 28°C for 1 h. The yeast cells were collected and ground in liquid nitrogen. Total RNA was extracted with TRIzol reagent. RNA integrity was assessed, and qualified RNA was used to construct a library. Transcriptomic sequencing was performed by Novogene Corporation (Beijing, China) using the Illumine NovaSeq platform. The reference genome and annotation files of C. albicans SC5314 were downloaded from NCBI (RefSeq accession number GCF_000182965.3). Hisat2 version 2.0.5 was applied to construct an index of the reference genome, and sequence alignment was performed between paired-end clean reads and the reference genome. The reads mapped to each gene were counted using FeatureCounts version 1.5.0-p3. The DESeq2 R package (1.20.0) was used to analyze differential gene expression, with thresholds of corrected *P* values < 0.05 and |log_2_(fold change)| > 1 (*P* values were corrected based on Benjamini and Hochberg’s approach). Kyoto Encyclopedia of Genes and Genomes (KEGG) enrichment analysis of DEGs was performed using the clusterProfiler R package (3.4.4).

### Time-killing kinetics.

Time-killing kinetics of different concentrations of peptide were conducted in the same way as the antimicrobial activity assay. The cell density of C. albicans was adjusted to approximately 5 × 10^4^ cells/mL. The suspensions of C. albicans were mixed with equal volumes of testing peptide (1×, 2× MBC) and incubated for 0, 1, 2, 4, 6, 8, or 10 h. The mixed suspensions were diluted and spread onto yeast extract peptone dextrose agar plates. After incubation for 24 h, the surviving colonies were counted. The experiments were repeated three times independently. When evaluating the effect of 3-MA on the candidacidal activity of Scyampcin_44–63_, C. albicans cells were pretreated with or without 10 mM 3-MA for 1 h. Different concentrations of Scyampcin_44–63_ were added and incubated for 30 min, 1 h, and 2 h. The final concentration contains 50% RPMI-MOPS (vehicle control). The mixed suspensions were diluted and spread onto plates. After incubation for 24 h, the surviving colonies were counted. The experiments were repeated twice independently.

### Antibiofilm activity assay.

Antibiofilm activity assay was performed as described previously, with some modifications (41). Briefly, 200 μL of a 2.5 × 10^6^ CFU/mL suspension of C. albicans in RPMI-MOPS was seeded in 96-well plates for 4 or 24 h, followed by washing with RPMI-MOPS to remove planktonic C. albicans. Fresh RPMI-MOPS with or without Scyampcin_44–63_ was added to the plate and further incubated for 24 h. The final concentration contains 50% RPMI-MOPS (vehicle control). After incubation, the planktonic C. albicans were removed by washing three times with sterile water. The biofilm was then stained with 100 μL of 0.1% crystal violet. Excessive stains were removed by washing the plate three times, 95% ethanol was used to dissolve the stain, and the absorbance was measured at 595 nm. The experiments were repeated three times independently.

### Evaluation of plasma membrane permeabilization.

A SYTOX Green nucleic acid stain (Invitrogen, USA) and LIVE/DEAD FungaLight yeast viability kit (Invitrogen, USA) were used to measure the cell membrane permeabilization induced by Scyampcin_44–63_. SYTOX Green is impermeable to intact membranes and exhibits more than 500-fold fluorescence enhancement after crossing compromised membranes and binding nucleic acids. C. albicans (2.5 × 10^6^ CFU/mL) was suspended in Hanks’ balanced salt solution (HBSS) containing 5 μM SYTOX Green, and an equal volume of Scyampcin_44–63_ at different concentrations was added to each well. The final concentration contains 50% HBSS (vehicle control). The fluorescence intensities (excitation [Ex], 485 nm; emission [Em], 525 nm) were constantly recorded every 5 min with a multimode microplate reader (Tecan, Switzerland). The experiments were repeated three times independently. The LIVE/DEAD FungaLight yeast viability kit contains 3.34 mM SYTO 9 green-fluorescent and 20 mM PI red-fluorescent nucleic acid stain. Syto 9 staining labels all yeast cells with intact or damaged membranes, while PI penetrates only yeast cells with damaged membranes, resulting in a reduced fluorescence intensity of Syto 9. A 2.5 × 10^6^ CFU/mL. C. albicans suspension in RPMI-MOPS was exposed to an equal volume of Scyampcin_44–63_ for 30 min. The final concentration contains 50% RPMI-MOPS (vehicle control). The samples were washed twice and suspended in HBSS containing Syto 9 and PI dye. The samples were transferred to poly-l-lysine slides and further stained for 15 min. Finally, the samples were imaged by confocal laser scanning microscopy (Zeiss, Germany). The experiments were repeated three times independently.

### Measurement of intracellular ROS.

Intracellular ROS were measured by using the cell-permeant reagent DCFH-DA (13). Once DCFH-DA enters the cell, DCFH-DA is hydrolyzed by cytoesterase to form DCFH, which is then rapidly oxidized to produce the strongly fluorescent product DCF. Briefly, fungal cells were pretreated with a DCFH-DA (Tocris Bioscience, UK) probe for 30 min at 28°C. After incubation, excessive probes were removed by washing twice with sterile PBS. Then, the fungi were resuspended in fresh RPMI-MOPS with or without different concentrations of Scyampcin_44–63_ or H_2_O_2_. The final concentration contains 50% RPMI-MOPS (vehicle control). The fluorescence intensities (Ex/Em = 485 nm/525 nm) were recorded using a multimode microplate reader (Tecan, Switzerland). The experiments were repeated three times independently.

### Mitochondrial membrane potential (ΔΨ_m_).

The effect of Scyampcin_44–63_ on the ΔΨ_m_ of C. albicans was determined with the lipophilic cationic dye JC-1 (5,5′,6,6′-tetrachloro-1,1′,3,3′-tetraethylimida-carbocyanine iodide, Solarbio) (42). Briefly, C. albicans (2.5 × 10^6^ cells/mL) in RPMI-MOPS were exposed to different concentrations of Scyampcin_44–63_ for 2 h at 28°C. The final concentration contains 50% RPMI-MOPS (vehicle control). After incubation, the suspensions were washed and stained with JC-1 at 37°C for 15 min. The excessive stains were washed off, and the samples were photographed on a confocal laser microscope (Zeiss, Germany). The experiments were repeated three times independently.

### TUNEL assay.

Apoptosis in C. albicans was detected by terminal deoxynucleotidyl transferase dUTP nick end labeling (TUNEL) assays as previously reported with some modifications (9). Briefly, C. albicans (2.5 × 10^6^ cells/mL) in RPMI-MOPS were treated with or without Scyampcin_44–63_ for 2 h at 28°C. The final concentration contains 50% RPMI-MOPS (vehicle control). The samples were washed twice with PBS and then fixed in 4% paraformaldehyde for more than 30 min at 4°C. The traces of paraformaldehyde were washed with PBS, and the cells were permeabilized with 0.3% Triton X-100 on ice for 30 min. TUNEL staining was performed according to the manufacturer’s instructions of the one-step TUNEL apoptosis assay kit (Beyotime, China). After staining, the samples were imaged with a confocal laser microscope. The experiments were repeated three times independently.

### Scanning electron microscopy (SEM).

SEM was applied to observe morphological changes in C. albicans caused by Scyampcin_44–63_. The final concentration contains 50% RPMI-MOPS (vehicle control). After incubation, fungal cells were fixed with 2.5% glutaraldehyde at 4°C for more than 90 min and washed three times before adhering to the surface of poly-l-lysine-coated glass slides. After 1 h of adhesion, the samples were dehydrated in graded ethanol (30% ethanol for 5 min, 50% ethanol for 5 min, and 70% ethanol for 10 min, where the above steps were performed on ice; and then 80% ethanol for 10 min, 95% ethanol for 15 min, and 100% ethanol twice for 15 min). Then all samples were critical point dried, coated with gold particles, and photographed with a field emission scanning electron microscope (FEI Quanta 650 FEG, USA). The experiments were repeated three times independently.

### Transmission electron microscopy (TEM).

TEM samples were prepared following the protocol reported by Liu and colleagues (43) with some modifications. Briefly, C. albicans (1.25 × 10^7^ cells/mL) were suspended in RPMI-MOPS, and an equal volume of peptide or sterile water (vehicle control) was added and followed by incubated for 1 h (28°C, 230 rpm). After treatment, the fungal cells were fixed with 2.5% glutaraldehyde and 0.5% paraformaldehyde. Glutaraldehyde and paraformaldehyde were diluted in 0.2 M phosphate buffer (pH of 6.8) and PIPES buffer (pH of 6.8), respectively. PIPES buffer contained 1 mM MgCl_2_, 1 mM CaCl_2_, and 0.1 M sorbitol. After fixation for 2 h at 4°C, the fungal cells were washed three times and pre-embedded in 2% agar. Then the specimens were postfixed with 1.5% KMnO_4_ for 2 h at 4°C and washed three times with sterile water. All samples were dehydrated in a graded acetone series (50%, 70%, 85%, 95%, 100%, 100%, and 100%) for 5 min each. After dehydration, all samples were transferred to graded ratios between epoxy resin and acetone (1:3, 1:1, and 3:1) for 1.5 h each, and all samples were incubated in pure epoxy resin overnight. Finally, all samples were sectioned into ultrathin sections, stained with uranyl acetate and lead citrate, and observed under a transmission electron microscope (Hitachi HT-7800, Japan). The experiments were repeated two times independently.

### Measurement of extracellular DNA concentration.

C. albicans (2.5 × 10^8^ cells/mL) in RPMI-MOPS were exposed to different concentrations of peptide or sterile water (vehicle control) and incubated for 2 h or 4 h. After incubation, the centrifuged supernatants were collected and measured using a NanoDrop 2000 spectrophotometer (Thermo Fisher Scientific, USA). The experiments were repeated three times independently.

### Cell cycle assay.

The DNA-specific fluorescent dye Sytox Green was applied to measure the DNA content. C. albicans (10^6^ cells) were collected after exposure to different concentrations of Scyampcin_44–63_ for 1 h. The final concentration contains 50% RPMI-MOPS (vehicle control). The samples were washed twice with 50 mM sodium citrate buffer and then fixed in cold 70% ethanol at 4°C for 12 h. The washing process was repeated for twice, and the cells were resuspended in buffer containing RNase A and incubated for 1 h at 37°C. SYTOX Green was then added and stained for 30 min (44). The DNA contents were measured with flow cytometer (Becton, Dickinson, USA). The data were analyzed using ModFit LT software (Verity Software House, USA). The experiments were repeated three times independently.

### Murine model of VVC.

Healthy C57BL/6J female mice (6 to 8 weeks old, 16 to 18 g) were purchased from GemPharmatech and acclimated for 1 week before the experiment. The mice were housed in individually ventilated cages (IVCs) on a 12-h light:12-h dark cycle at room temperature and provided access to sterile food and water *ad libitum*. The murine model of Candida vaginitis was constructed following a previously described study with some modifications (35, 45). Briefly, each mouse was injected subcutaneously with 100 μL of sesame oil containing 200 μg of estradiol valerate (Sigma-Aldrich, Unite States). Three days later, each mouse was inoculated intravaginally with 10 μL of C. albicans (2.5 × 10^7^ CFU/mL). Twenty-four hours postinfection, mice received vaginal perfusion of 10 μL phosphate-buffered saline or different concentrations of Scyampcin_44–63_ (12 or 48 μM/group) or 100 μg/mL fluconazole every 12 h. After 3 consecutive days, the vagina of each mouse was rinsed with 200 μL of PBS. The lavage fluid was diluted with PBS and then spread on Sabouraud’s dextrose agar plates containing 1.25 μg/mL chloramphenicol (Solarbio, China) for fungal quantification. The aforementioned animal experiments were performed according to national guidelines and approved by the Laboratory Animal Management and Ethics Committee of Xiamen University (XMULAC20220048). The experiments were performed twice independently.

For histology, the vaginal tissues were fixed with 4% formaldehyde, and then paraffin-embedded vaginal tissues were obtained. Sections were cut from the embedded tissues and subjected to hematoxylin and eosin staining (3).

### Statistical analysis.

All data are represented as the means ± standard error of the mean. Two-tailed Student’s *t* test, one-way analysis of variance (ANOVA) or two-way ANOVA was used to analyze the differences between different groups. Figure 2 and 7 used one-way ANOVA, Fig. 3 used two-way ANOVA, and the other figures used Student’s *t* test. *P* values < 0.05 were considered significant.

The DESeq2 R package (1.20.0) was used to analyze transcriptomic data for differential gene expression with thresholds of corrected *P* values < 0.05 and |log_2_(fold change)| > 1 (*P* values corrected base on Benjamini and Hochberg’s approach).

### Data availability.

All relevant data are within the article and its supporting information files. The full-length cDNA sequence of Scyampcin and transcriptomic data of C. albicans are both available on NCBI database (GenBank accession number MW388710; BioProject number PRJNA906206).
