# Supplementary material for: Newly Discovered Antimicrobial Peptide Scyampcin44–63 from Scylla paramamosain Exhibits a Multitargeted Candidacidal Mechanism In Vitro and Is Effective in a Murine Model of Vaginal Candidiasis
Source: Antimicrob Agents Chemother. 2023 May 10;67(6):e00022-23. doi: 10.1128/aac.00022-23 (PMC10269043; doi:10.1128/aac.00022-23)
Supplement: Supplemental file 1 — Supplemental material. Download aac.00022-23-s0001.docx, DOCX file, 11.4 MB [file aac.00022-23-s0001.docx]

### TABLE S1 Primer sequences.

| Primers | Sequence (5′–3′) |
| --- | --- |
| Scyampcin-5'-R1 | CTGTTTCCTTGGATGTACTGGTGTC |
| Scyampcin-5'-R2 | TGTAGCAGTCCAGGCAAGGGGCAT |
| Scyampcin-3'-F1 | CTTCCTTGAGCCAACGCCAAACACC |
| Scyampcin-3'-F2 | GATGCCCCTTGCCTGGACTGCTACA |
| Long primer | CTAATACGACTCACTATAGGGCAAGCAGTGGTATCAACGCAGAGT |
| NUP | AAGCAGTGGTATCAACGCAGAGT |

### TABLE S2 Website of related database or analysis software.

| Database or analysis software | Website |
| --- | --- |
| NCBI | <https://www.ncbi.nlm.nih.gov/> |
| Expasy | <https://web.expasy.org/compute_pi/> |
| PSIPRED 4.0 | <http://bioinf.cs.ucl.ac.uk/psipred/> |
| I-TASSER | <https://zhanglab.ccmb.med.umich.edu/I-TASSER/> |
| CAMP_R3_ | (<http://www.camp3.bicnirrh.res.in/index.php>) |

### TABLE S3 Primer sequences for RT-qPCR.

| Primers | Sequence (5′–3′) |
| --- | --- |
| ATG15-for | TGGTGACCGATAAAGGCTGGAG |
| ATG15-rev | GCTGCTGCTTCGTAGTGGATTG |
| CDC28-for | CGTCAAGAAAAAGTCGGAGAAG |
| CDC28-rev | CACCTTCATCTTCTGATTCTAA |
| CHS2-for | TCCGTTCCAAGAAACTCACTCG |
| CHS2-rev | TTACTGCTGTATATCTCATAAAGGC |
| CLB2-for | TCACCACCTCAAGTCTCTGTAA |
| CLB2-rev | GTAGAGGGGCATTGTCTCCACC |
| CLB4-for | GAAACACAATCACATACACAGG |
| CLB4-rev | TTTATTATCGCATTATTCCACA |
| ERG11-for | TTTAGTTTCTCCAGGTTATGCTCA |
| ERG11-rev | ATTAGCTTTGGCAGCAGCAGTA |
| ERG1-for | AGAATGTGTTAACGGGCCAATT |
| ERG1-rev | ATGGTTGAATAACAACATTGGGAAT |
| ERG3-for | GGCCATCTGTTTACAAAGTGTTACA |
| ERG3-rev | TGAGAAGCAAATGGAGTACAAACAA |
| ERG6-rev | CCAGGACCACCTACACCACA |
| ERG6-for | TGGTTGGGGTTCTTCATTCC |
| KRE1-for | CCTTGCGGCAGATAAAACGT |
| KRE1-rev | GCATCAGTACCTGTGACCCATACT |
| MNN1-for | ATTATTACAACACGGAAAGCCA |
| MNN1-rev | ATGACAAGGCAGATTTCGGTGG |
| PCL5-for | AAATCATTACCACACGCTAACTTG |
| PCL5-rev | GTGATGGTTTGGAGATAAAGTAGA |
| 18S rRNA-for | AATTACCCAATCCCGACAC |
| 18S rRNA-rev | TGCAACAACTTTAATATACGC |


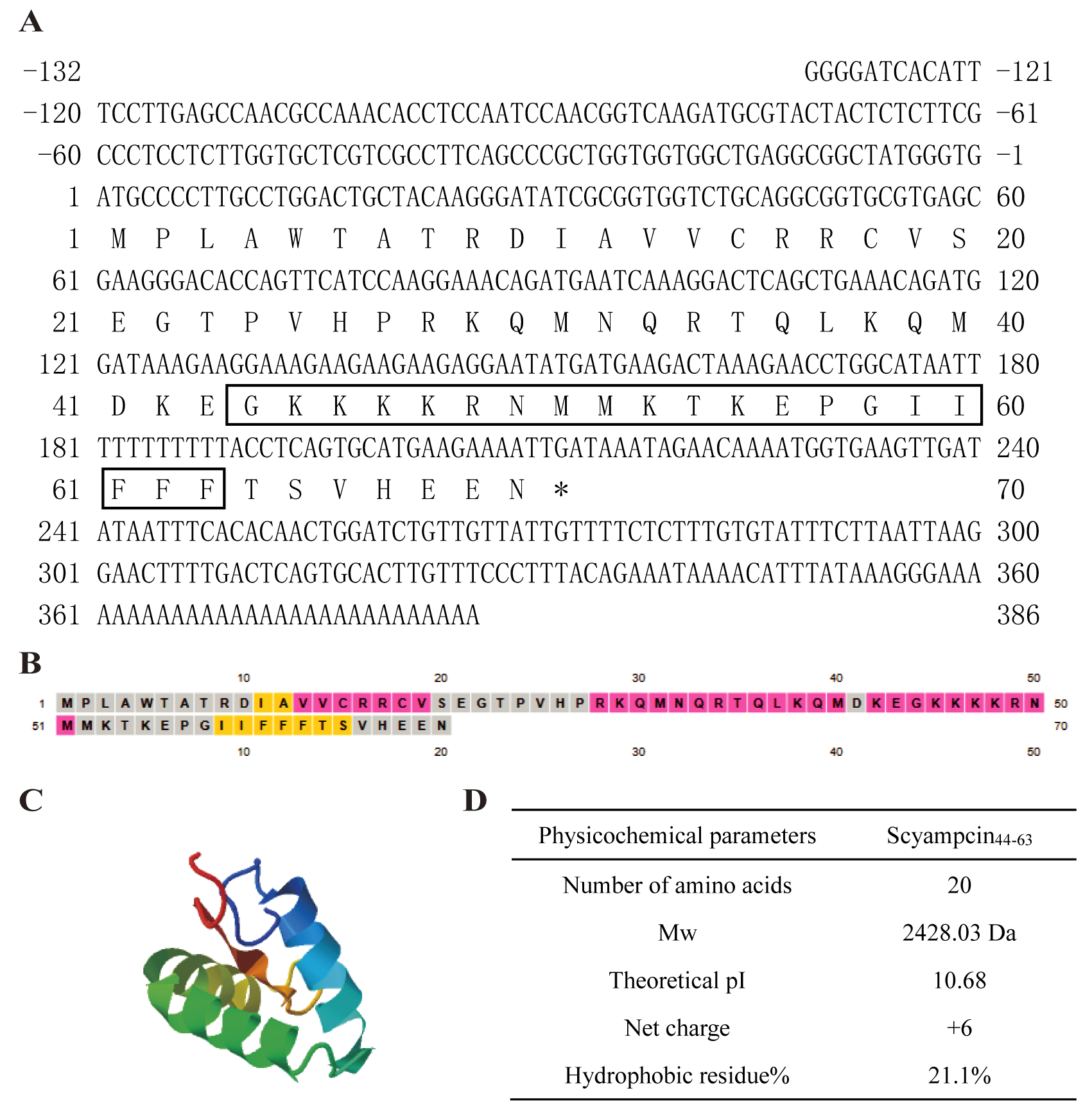


**Fig. S1** **Bioinformatics analysis. A)** Full-length cDNA and deduced amino acid sequence of Scyampcin. Asterisk indicates the stop codons, sequence of Scyampcin_44-63_ was shown in rectangle. **B)** The secondary structure was predicted by PSIPRED. The yellow regions indicate strand, pink regions indicate helix and gray regions indicate coil. **C)** Tertiary structural model was constructed by I-TASSER server. **D)** The properties of Scyampcin_44-63_.


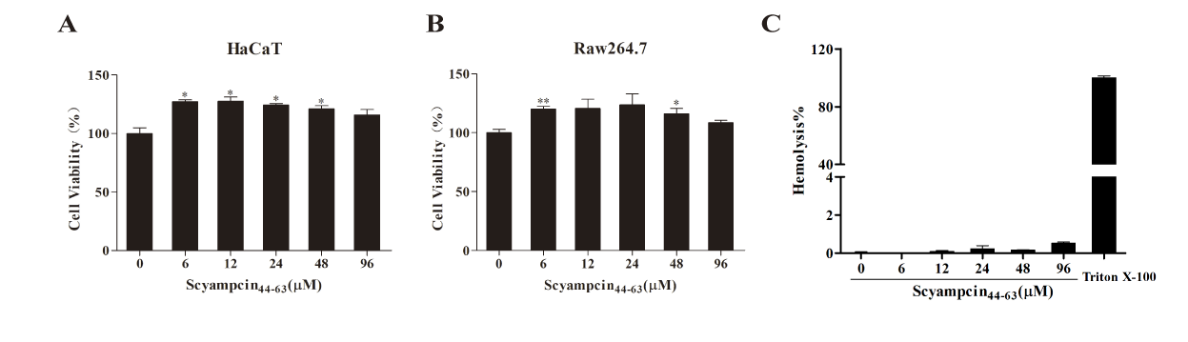


**Fig. S2 Cytotoxicity and hemolysis of Scyampcin_44-63_.** **A-B)** The effects of Scyampcin_44-63_ on cells viability of HaCaT and Raw264.7. **C)** The hemolysis activity of Scyampcin_44-63_ to mouse erythrocytes. Treatment of 1% TritonX-100 was used as a positive control. Representative results of three repeats, and error bars represent standard errors of the means (n = 3).

### TABLE S4 The up-regulated genes response to Scyampcin_44-63_.

| **Gene name** | **Description** | **log_2_Fold_change** | **Corrected *p* values** |
| --- | --- | --- | --- |
| **Cell wall synthesis** | |  |  |
| EXG2 | Glucan 1,3-β-glucosidase 2 | 2.85 | 3.48 × 10^-55^ |
| PIR1 | β-1,3-glucan linked protein | 3.13 | 4.58 × 10^-184^ |
| PHR2 | β-1,3-glucan transglycosylases | 1.60 | 3.22 × 10^-120^ |
| PHR1 | β-1,3-glucan transglycosylases | 1.48 | 1.03 × 10^-77^ |
| KRE1 | β-1,6-glucan synthase | 2.97 | 2.50 × 10^-112^ |
| KRE62 | β-1,6-glucan biosynthesis | 2.16 | 1.12 × 10^-08^ |
| SKN1 | N-glycosylated type II membrane protein | 1.17 | 6.70 × 10^-18^ |
| CHS2 | Chitin synthase | 2.72 | 7.57 × 10^-99^ |
| BMT7 | β-mannosyltransferase | 2.86 | 2.64 × 10^-106^ |
| CRH11 | Chitin transglycosylases | 3.00 | 2.95 × 10^-141^ |
| MNN1 | α-1,3-mannosyltransferase | 2.27 | 3.78 × 10^-67^ |
| MNN15 | α-1,3-mannosyltransferase | 1.60 | 4.68 × 10^-35^ |
| MNN13 | α-1,3-mannosyltransferase | 1.11 | 1.10 × 10^-22^ |
| VPS34 | Phosphatidylinositol 3-kinase | 1.28 | 9.18× 10^-40^ |
| **CWI pathway** | |  |  |
| RLM1 | Transcription factor | 1.37 | 7.19 × 10^-32^ |
| CEK1 | Extracellular signal-regulated kinase 1 | 1.22 | 3.97 × 10^-20^ |
| MKC1 | Mitogen-activated protein kinase | 1.06 | 1.48 × 10^-24^ |
| **Anti-oxidative stress** | |  |  |
| SOD5 | Cu-only superoxide dismutase 5 | 5.50 | 1.94 × 10^-263^ |
| SOD6 | Superoxide dismutase [Cu-Zn] 6 | 1.92 | 7.37 × 10^-36^ |
| CAT1 | Peroxisomal catalase | 1.39 | 1.73 × 10^-26^ |
| GPX1 | Glutathione peroxidase | 1.13 | 5.83 × 10^-10^ |
| GRX1 | Glutaredoxin | 1.05 | 2.38 × 10^-06^ |
| YHB5 | Nitric oxide dioxygenase | 2.57 | 8.85 × 10^-47^ |
| YHB4 | Nitric oxide dioxygenase | 1.44 | 2.78 × 10^-43^ |
| **Apoptosis** | |  |  |
| MCA1 | Metacaspase1 | 1.08 | 5.22 × 10^-34^ |
| **ER stress** |  |  |  |
| HAC1 | Transcription factor | 1.60 | 1.12 × 10^-43^ |

Corrected *p* values were adjusted using the Benjamini & Hochberg method, which helped to better control the false positive rate of the multiple hypothesis test.

### The Cell Wall Synthesis Related Genes.

β-1,3-glucan biosynthesis related genes (*EXG2,* *PIR1*, *PHR2* and *PHR1*) were up-regulated (1.48-fold to 3.13-fold), *PIR1* was up-regulated mostly. β-1,6-glucan biosynthesis associated genes (*KRE1*, *KRE62* and *SKN1*) were up-regulated (1.17-fold to 2.97-fold). Chitin synthase *CHS2* and chitin transglycosylases *CRH11* were up-regulated 2.72-fold and 3.00-fold, respectively. β-mannosyltransferase *BMT7* and α-1,3-mannosyltransferase (*MNN1*, *MNN15*, *MNN13*) were up-regulated 1.11-fold to 2.86-fold. Phosphatidylinositol 3-kinase *Vps34* possesses cellular transport, may participates in production and delivery of cell wall building enzymes to the cell periphery, was increased 1.28-fold (**Table S4**).

### CWI Pathway Related Genes

*MKC1* encodes mitogen-activated protein (MAP) kinase, which regulates CWI pathway and induces cell wall remodeling response(1). *RLM1* is one of the transcription factors involves in the CWI pathway and *CEK1* participates in cell wall construction. After treatment of Scyampcin_44-63_, *RLM1*, *CEK1* and *MKC1* were up-regulated 1.37-fold, 1.22-fold and 1.06-fold, respectively (**Table S4**).

### Oxidative Stress Responsive Genes.

Seven anti-oxidative stress genes (*SOD5*, *SOD6*, *CAT1*, *GPX1*, *GRX1*, *YHB5*, *YHB4*) were up-regulated (1.05-fold to 5.50-fold). Among these genes, cell surface superoxide dismutase *SOD5* was up-regulated mostly. Superoxide dismutases *SOD5* and *SOD6* convert superoxide to oxygen and less toxic hydrogen peroxide(H_2_O_2_), catalase *CAT1* and glutathione peroxidases *GPX1* detoxicate H_2_O_2_ to water and oxygen. The glutaredoxin *GRX1*, which belongs to glutathione system, is responsible for repairing oxidatively damaged protein thiols. Nitric oxide-detoxifying flavohemoglobin gene YHB4 and YHB5 were up-regulated 1.44-fold and 2.57-fold, respectively (**Table S4**).

### ER Stress and Apoptosis Related Genes.

Transcription factor HAC1 plays a major role in stress-related transcriptional response(2) , which is required for ER stress resistance in *C. albicans*(3). Metacaspase *MCA1* has been proven to participate in oxidative stress-induced cell death(4).After exposed to Scyampcin_44-63_, *HAC1* and *MCA1* were up-regulated 1.60-fold and1.08-fold, respectively (**Table S4**).

### Ergosterol Biosynthesis Related Genes

In response to Scyampcin_44-63_ treatment, ergosterol biosynthesis related genes (*ERG3*, *ERG6*, *ERG13*, *ERG2*, *ERG5*, *ERG25*, *ERG251*, *ERG1*, *ERG20*, *ERG12*, *ERG24*, *ERG11*, *MVD*, *IDI1*, *MET6*, *SAM2*, *COQ5*, *CYB5*) were down-regulated (1.03-fold to 2.48-fold) (**Table S5**). The mostly down-regulated was homocysteine methyltransferase-encoding gene, *MET6*. Squalene epoxidase ERG1 is considered to be a rate-limiting enzyme in steroid biosynthesis, which catalyzes the stereospecific oxidation of squalene to (S)-2,3-epoxysqualene. The lanosterol 14-alpha-demethylase ERG11 catalyzes C14-demethylation of lanosterol to produce 4,4'-dimethyl cholesta-8,14,24-triene-3-beta-ol, which is critical for ergosterol biosynthesis. Cytochrome *b*5 *CYB5* is involved in sterol biosynthesis in yeast, probably as a cofactor, and support *ERG11* function by acting as an alternative electron carrier(5). *CYB5* and *ERG11* were decreased 1.21-fold and 1.03-fold, respectively (**Table S5**). Sterol C-24 methyltransferase (encoded by *ERG6*) is involved the sub pathway that synthesizes ergosterol from zymosterol, which requires S-adenosylmethionine synthesized from methionine by the S-adenosylmethionine synthetase (*SAM*)(5). Both *ERG6* and *SAM2* genes were down-regulated 2.06-fold and 2.21-fold, respectively (**Table S5**). *COQ5* is involved in the pathway ubiquinone biosynthesis, which starts with converting isopentenyl pyrophosphate into dimethyl allyl-pyrophosphate, both of these pyrophosphates are intermediates of the ergosterol biosynthesis pathway(5). *COQ5* was decreased 1.47-fold upon Scyampcin_44-63_ treatment (**Table S5**).

### TABLE S5 The down-regulated genes response to Scyampcin_44-63_.

| **Gene name** | **Description** | **log_2_Fold_change** | **Corrected *P*-value** |
| --- | --- | --- | --- |
| **Ergosterol Biosynthesis** | | | |
| ERG3 | C-5 sterol desaturase | -2.35 | 3.59 × 10^-54^ |
| ERG6 | Sterol 24-C-methyltransferase | -2.06 | 6.45 × 10^-55^ |
| ERG13 | Hydroxymethylglutaryl-coa synthase | -1.98 | 1.16 × 10^-111^ |
| ERG2 | C-8 sterol isomerase | -1.75 | 4.25 × 10^-46^ |
| ERG5 | C-22 sterol desaturase | -1.58 | 1.38 × 10^-98^ |
| ERG25 | Methylsterol monooxygenase | -1.57 | 6.43 × 10^-22^ |
| ERG251 | Erg251p | -1.41 | 6.14 × 10^-31^ |
| ERG1 | Squalene epoxidase | -1.27 | 1.70 × 10^-65^ |
| ERG20 | Farnesyl pyrophosphate synthase | -1.10 | 5.54 × 10^-26^ |
| ERG12 | Mevalonate kinase | -1.10 | 5.00 × 10^-28^ |
| ERG24 | Δ (14)-sterol reductase | -1.06 | 5.29 × 10^-28^ |
| ERG11 | Lanosterol 14-α-demethylase | -1.03 | 3.66 × 10^-33^ |
| MVD | Diphosphomevalonate decarboxylase | -1.17 | 2.84 × 10^-38^ |
| IDI1 | Isopentenyl-diphosphate Δ-isomerase | -1.92 | 2.28 × 10^-41^ |
| MET6 | Homocysteine S-methyltransferase | -2.48 | 1.27 × 10^-141^ |
| SAM2 | S-Adenosylmethionine synthetase 2 | -2.21 | 1.50 × 10^-27^ |
| COQ5 | 2-methoxy-6-polyprenyl-1,4-benzoquinol methylase | -1.47 | 9.57 × 10^-26^ |
| CYB5 | Cytochrome *b*5 | -1.21 | 1.76 × 10^-40^ |

Corrected *p* values were adjusted using the Benjamini & Hochberg method, which helped to better control the false positive rate of the multiple hypothesis test.

**
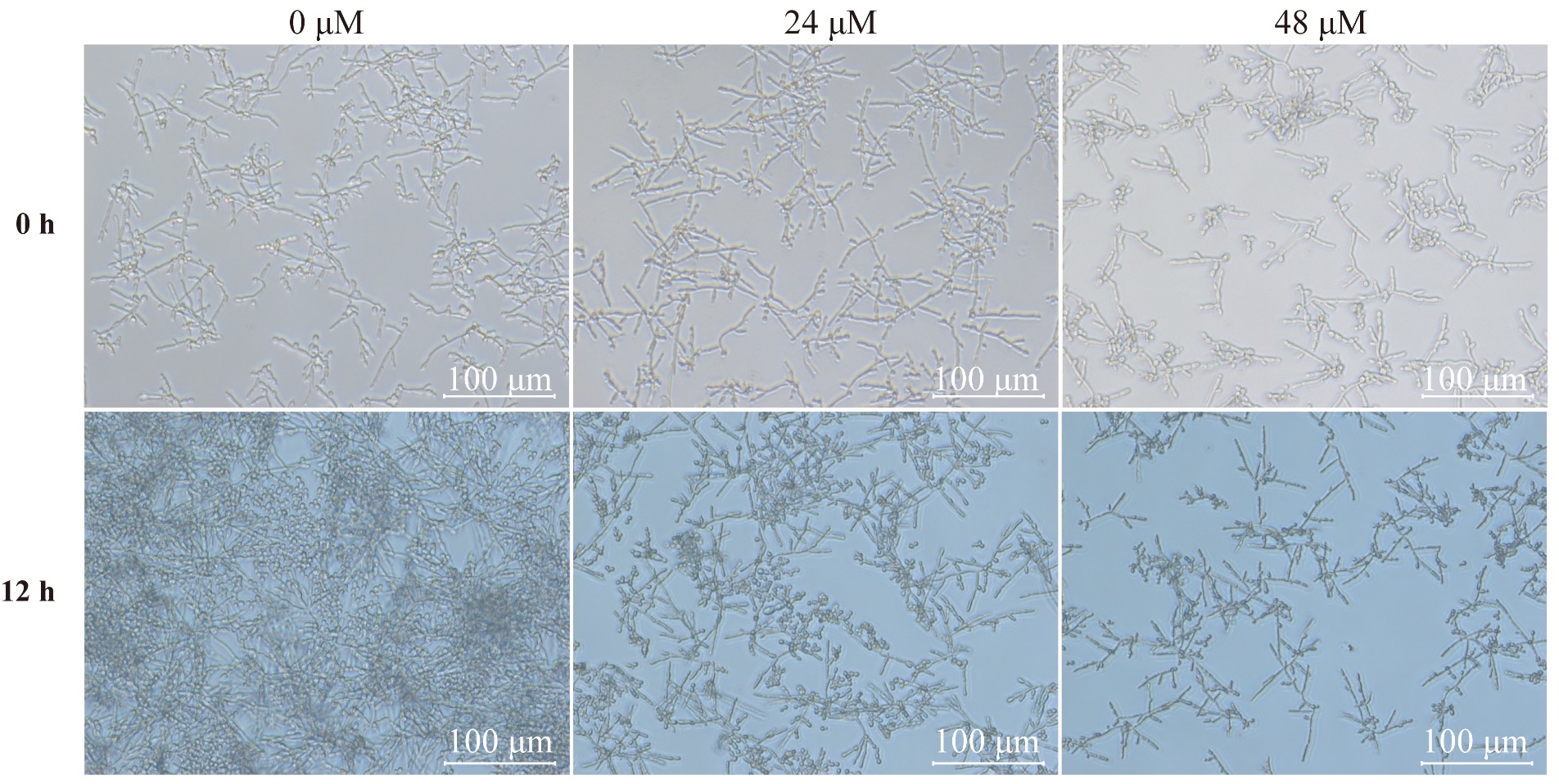
**

Fig. S3 **Efficacy of Scyampcin_44-63_ against hyphal forms of *C. albicans***. *C. albicans* (2.5 × 10^5^ cells/mL) was cultivated at 37 ℃ for 5 h until hyphal formed, and then Scyampcin_44-63_ was added and incubated for another 12 h (the time in the image was the incubation time of *C. albicans* with Scyampcin_44-63_), and photographed. Representative images of three repeats.


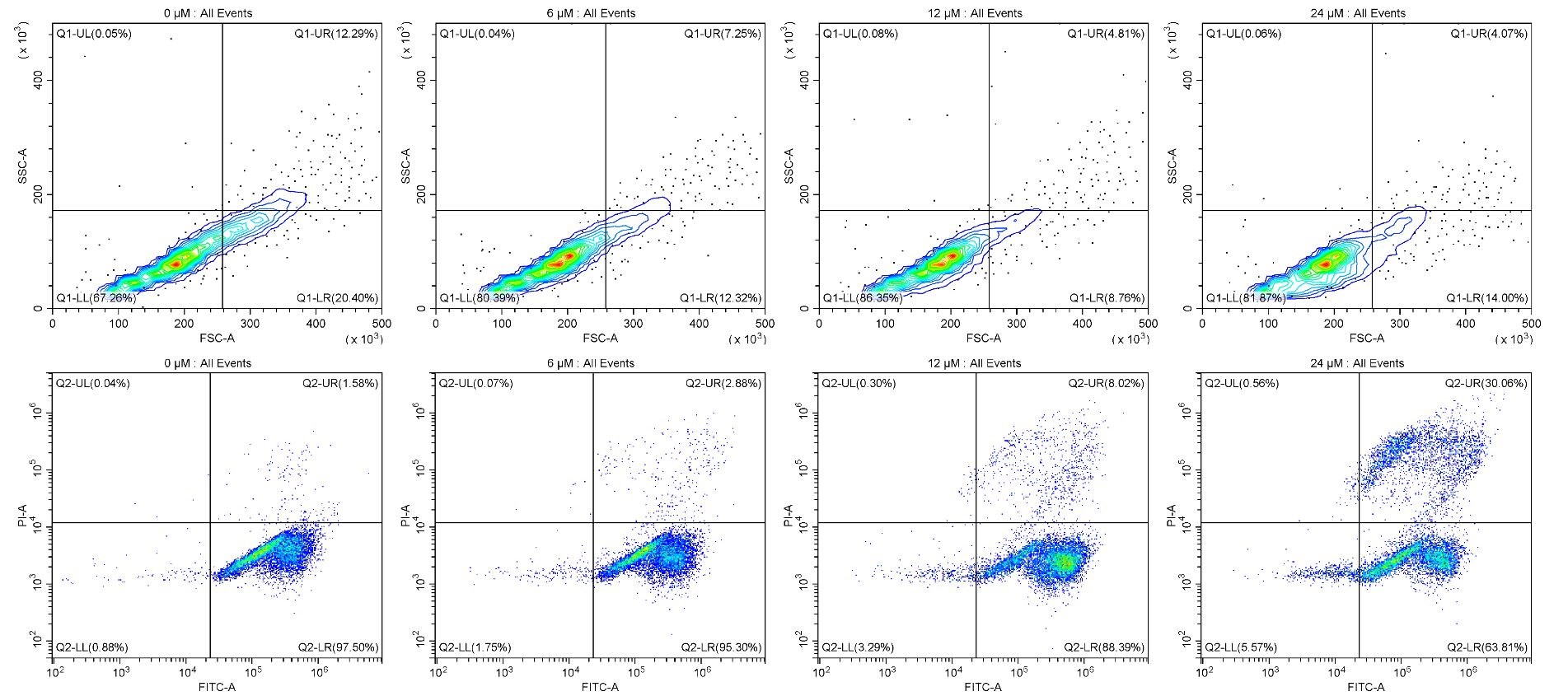


**Fig. S4 Scyampcin_44-63_ induces cell shrinkage and membrane permeability in *C. albicans.*** *C. albicans* (5 × 10^6^ cells/mL) were incubated with Scyampcin_44-63_ for 15 min, and then stained with PI/Syto 9 and measured by flow cytometry. The upper figure represents flow cytometric contour plot analysis of *C. albicans*. FSC is an indicator of cell size, and SSC is an indicator of cell granularity. PI indicates the compromised membrane, and FITC represents Syto9 channel, Syto 9 indicates live cells.

### TABLE S6 Effect of exogenous ergosterol on the MICs of Scyampcin_44-63_ and AMB on *C. albicans*.

| Ergosterol(μg/ml) | *C. albicans* MIC | |
| --- | --- | --- |
|  | Scyampcin_44-63_ (μM) | AMB (μg/ml) |
| 0 | 3-6 | 0.625-1.25 |
| 50 | 3-6 | 2.5-5 |
| 100 | 6-12 | 2.5-5 |
| 200 | 6-12 | 5-10 |


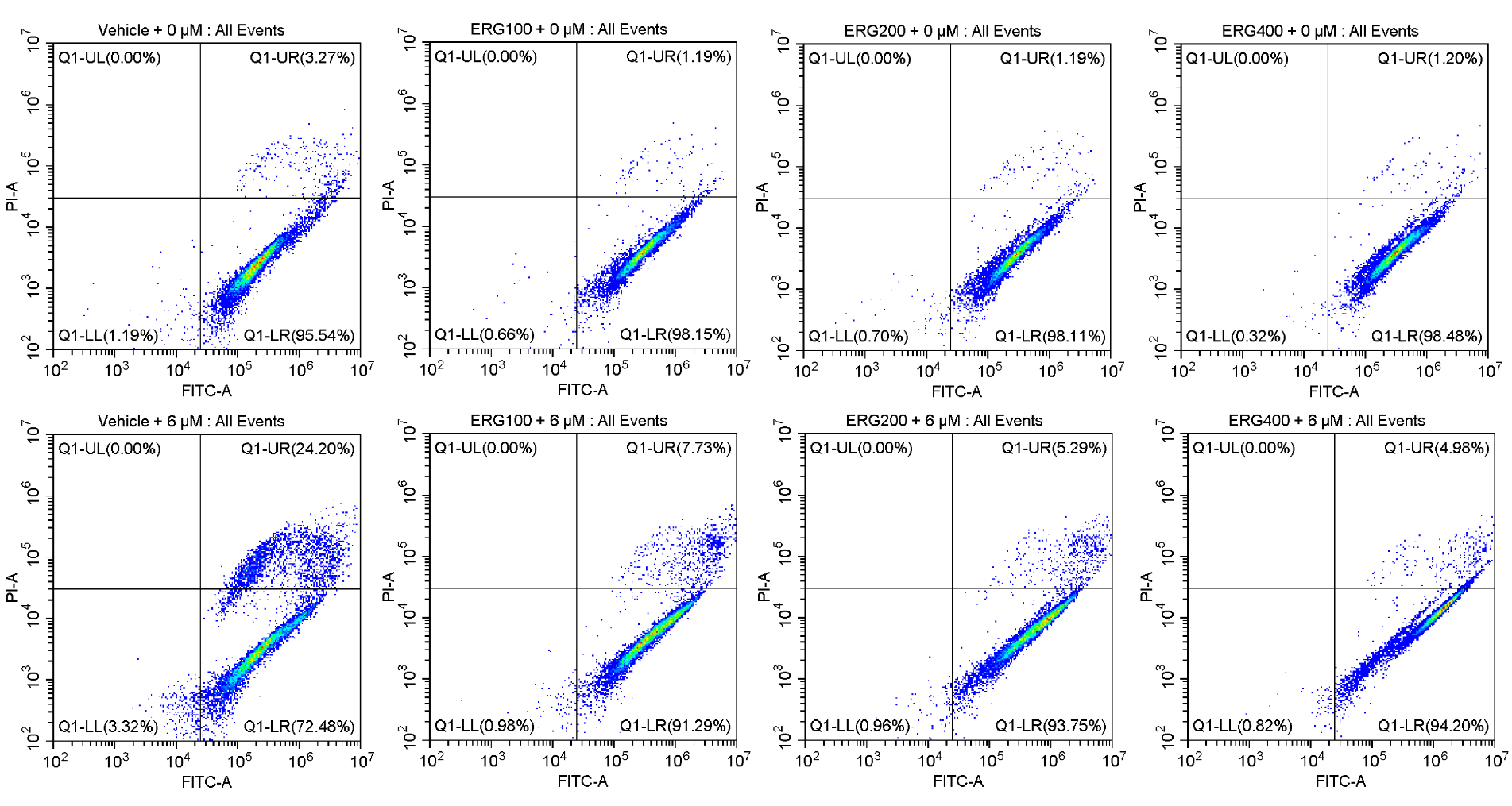


**Fig. S5 Ergosterol influenced the efficacy of Scyampcin_44-63_ against *C. albicans*.** 12 μM Scyampcin_44-63_ were pretreated with or without (as a vehicle control) different concentrations of ergosterol (100, 200 and 400 μg/mL, in representative images were shorted as ERG100, ERG200 and ERG400, respectively) for 6 h, and then incubated with equal volume suspensions of *C. albicans* (2.5 × 10^6^ cells/mL) for another 10 min (The final concentration of Scyampcin_44-63_ was 6 μM). The suspensions were washed with HBSS for two time, stained with PI/Syto 9, and measured with flow cytometer. Representative images of three repeats





**Fig. S6 Tissue damage detection by LDH assay.** In murine *C. albicans* vaginitis, mice were treated with PBS, 12 μM Scyampcin_44-63_, 48 μM Scyampcin_44-63_ or 100 μg/mL Fluconazole for 3 days (at intervals of 12 h) at 24 h post-infection. The lavage fluids were collected for LDH assay. The lysate of lavage fluid of PBS group was used as a positive control. ** indicates p <0.01. Representative results of two repeats, and error bars represent standard errors of the means (n = 4).

1. Heilmann CJ, Sorgo AG, Mohammadi S, Sosinska GJ, de Koster CG, Brul S, de Koning LJ, Klis FM. 2013. Surface stress induces a conserved cell wall stress response in the pathogenic fungus Candida albicans. Eukaryot Cell 12:254-64.

2. Lee J, Kim J-G, Lee H, Lee TH, Kim K-Y, Kim H. 2021. Antifungal Activity of 1,4-Dialkoxynaphthalen-2-Acyl Imidazolium Salts by Inducing Apoptosis of Pathogenic Candida spp. Pharmaceutics 13.

3. Wimalasena TT, Enjalbert B, Guillemette T, Plumridge A, Budge S, Yin Z, Brown AJ, Archer DB. 2008. Impact of the unfolded protein response upon genome-wide expression patterns, and the role of Hac1 in the polarized growth, of Candida albicans. Fungal Genet Biol 45:1235-47.

4. Cao Y, Huang S, Dai B, Zhu Z, Lu H, Dong L, Cao Y, Wang Y, Gao P, Chai Y, Jiang Y. 2009. Candida albicans cells lacking CaMCA1-encoded metacaspase show resistance to oxidative stress-induced death and change in energy metabolism. Fungal Genet Biol 46:183-9.

5. De Backer MD, Ilyina T, Ma XJ, Vandoninck S, Luyten WH, Vanden Bossche H. 2001. Genomic profiling of the response of Candida albicans to itraconazole treatment using a DNA microarray. Antimicrob Agents Chemother 45:1660-70.
